# Integrating GPS and Accelerometry to Capture Life-Space Mobility in Parkinson’s Disease

**DOI:** 10.3390/s26082480

**Published:** 2026-04-17

**Authors:** Caitríona Quinn, Hanna Johansson, Camilla Malinowsky, Breiffni Leavy

**Affiliations:** 1 Division of Physiotherapy, Department of Neurobiology, Care Sciences and Society, Karolinska Institutet, Alfred Nobels Allé 23, Huddinge, 141 83 Stockholm, Sweden; hanna.johansson.1@ki.se (H.J.); breiffni.leavy@ki.se (B.L.); 2 Stockholm Sjukhem Foundation, Mariebergsgatan 22, 112 19 Stockholm, Sweden; 3 Theme Women’s Health and Allied Health Professionals, Medical Unit Occupational Therapy & Physiotherapy, Karolinska University Hospital, 141 57 Stockholm, Sweden; 4 Division of Occupational Therapy, Department of Neurobiology, Care Sciences and Society, Karolinska Institutet, Alfred Nobels Allé 23, Huddinge, 141 83 Stockholm, Sweden; camilla.malinowsky@ki.se

**Keywords:** Parkinson’s disease, life-space mobility, global positioning systems, accelerometry, physical activity

## Abstract

Mobility is key to independent living, and life-space mobility (LSM) describes the extent to which a person moves within and beyond their home. Restricted LSM may limit participation in life. Parkinson’s disease (PD) can influence LSM through changes in physical function and social engagement. This study investigated LSM in people with mild-to-moderate PD using self-reported and objective measures, examining agreement between measures and relationships with physical activity and quality of life. The participants completed the Life-Space Assessment (LSA) and wore an accelerometer and GPS monitor for seven days. The accelerometer and GPS data were processed to derive GPS life-space and physical activity variables. The participants (n = 11) demonstrated variability in GPS-derived measures of LSM despite high levels of self-reported LSM (median 82). The participants spent approximately two thirds of wear time in close proximity to the home, where most activity time was sedentary. Higher levels of moderate-to-vigorous physical activity and step counts were observed beyond the home. Self-reported health-related quality of life showed weak associations with LSM. These findings suggest that while the LSA captures overall mobility, GPS data provides complementary insight into location-specific activity and individual movement patterns.

## 1. Introduction

Mobility is a core requirement for independence, participation, and quality of life in individuals with chronic health conditions. The term life-space mobility (LSM) is used to describe the pattern of areas that a person moves within, extending from the location where a person sleeps to beyond their town or geographic region [1,2]. It provides valuable information regarding the movement, independence, and abilities of a person to participate in daily life, and can reflect mobility in the context of an individual’s real-world experiences and priorities, making it a person-centered indicator of participation in daily life. LSM reflects an individual’s involvement in the everyday environment, aligning with the participation domain of the International Classification of Functioning, Disability, and Health (ICF) [3]. Research has demonstrated a decline in LSM with advancing age [4], and restricted life space is associated with numerous negative health outcomes including increased frailty [5,6,7], falls [8,9], fear of falling [10], reduced health-related quality of life [7,11], reduced physical activity [12,13], and progression of cognitive decline [14,15]. These challenges may be particularly relevant to clinical populations characterized by mobility impairment, such as people living with Parkinson’s disease (PD).

PD is a progressive neurodegenerative disease, characterized by motor and non-motor symptoms that can lead to limitations in functional capacity, affecting activities of daily living and participation in everyday life [16]. Social withdrawal is common in people with PD, which can arise from both movement difficulties and stigma and restrictions associated with motor and cognitive symptoms. The resulting isolation may have a detrimental impact on health and quality of life [17]. Levels of physical activity in people with PD have been associated with performance of activities of daily living, functional mobility, and quality of life [18]. Considering that people with PD often demonstrate reduced physical activity levels [19,20,21,22], this may contribute to participation restrictions and barriers to participation reported in this population [23,24,25,26].

The University of Alabama Life-Space Assessment (LSA) was developed to measure mobility in the previous four weeks at five environmental levels, from within the home to beyond the town [1]. Frequency of visits to, and independence at, each level is assessed. A composite score sums all levels, with possible scores ranging from 0 to 120, where higher scores indicate greater LSM. The LSA is a valid and reliable measure of LSM and has been translated into several languages, including Swedish, with psychometric properties established in community-dwelling older adults [27,28]. The LSA has also been used in several studies to assess LSM in people with PD [29,30,31,32,33].

The LSA is a self-reported measure of LSM and so may be influenced by recall bias. The use of movement sensors and the Global Positioning System (GPS) can provide real-world mobility data, giving detailed insights into the specific movements undertaken and places visited. Additionally, continuous GPS recordings can facilitate the collection of large amounts of real-time mobility data that is not dependent on recall. GPS monitors are becoming an increasingly common method of assessing mobility in older adult populations [34,35,36,37,38,39,40]; however, there is limited research regarding people with PD [29,30,41].

GPS monitoring can be used to understand the geographic extent of movement, whereas accelerometers can quantify the movement intensity. The combination of these digital methods may offer further insights into mobility and health beyond self-reported outcomes. Combining these sensors may enable a more in-depth assessment of LSM, capturing not only where an individual travels but also the activity associated with different locations. The combined use of accelerometers and GPS monitors appears feasible and has been reported in several studies [15,35,39,42]; however, there is a lack of consistency and guidance in the analysis and reporting of these methods.

Research on LSM in PD has largely relied on self-reported measures, which are subject to recall bias [29,31,32,33]. Integrating objective measurements to supplement self-reported measures offers the potential to develop a more comprehensive view of LSM and daily activity in people with PD. This can help to inform strategies to maintain participation and mobility in this cohort. While self-reported measures provide valuable insights into perceived mobility, they may not always align with objectively collected mobility data [36], highlighting the need to further understand the agreement between these measures. The present study aims to examine LSM in people with mild-to-moderate PD using self-reported and objective measures, to investigate the agreement and differences between these methods, and to investigate physical activity and health-related quality-of-life outcomes in relation to LSM in this cohort.

## 2. Materials and Methods

### 2.1. Study Participants

A sub-group of participants recruited in the Support for home Training using Ehealth in Parkinsons diseaSe (STEPS) randomized controlled trial (RCT), was included in this study. Eligibility criteria for the STEPS trial, a 10-week home exercise intervention, included a diagnosis of idiopathic PD for ≥6 months, age ≥ 50 years, ability to walk indoors continuously for ≥6 min without a walking aid, ≤2 falls in the last month, stable PD medication habits for ≥3 months, and a score of ≥21 on the Montreal Cognitive Assessment (MoCA). Participants were excluded if they had any other neurological or orthopedic disorder that impeded gait or balance [43]. The study was approved by the Swedish Ethical Review Authority (Dnr: 2022-02979-01, 2023-07565-02 and 2023-00717-02).

### 2.2. Demographic Information and Outcome Measures

Information regarding age, gender, years since PD diagnosis, years of education, marital status, housing type, falls history, and mobility aid use was collected. The Hoehn and Yahr scale was used to determine motor symptom progression and level of disability [44]. The Five Times Sit-to-Stand (5xSTS) test was performed once to assess lower extremity functional strength [45]. The Mini Balance Evaluation Systems Test (Mini-BESTest) was used to assess balance performance [46]. Self-reported measures were completed including the Walk-12 to assess walking ability [47], the Activity-specific Balance Confidence scale (ABC) to assess balance confidence [48], the Euroqol 5-Dimension scale (EQ-5D) to assess health-related quality of life [49], and the Parkinson’s Disease Questionnaire-39 (PDQ-39) [50] to assess disease-specific health-related quality of life. The assessments were carried out during the on stage of levodopa medication, and the order of the tests was the same for all participants. Demographic and outcome measure data was collected in a primary care rehabilitation setting in Stockholm, Sweden.

### 2.3. Life-Space Assessment

The LSA is a 15-item self-reported measure of LSM. It assesses five life-space levels over the previous four weeks: outside the bedroom but within the home, outside the home, within the neighborhood, beyond the neighborhood but within the town, and beyond the town [2]. The LSA assesses frequency and independence at each of the life-space levels. A composite score is calculated by totaling all the life-space levels achieved and multiplying by the frequency and independence values. LSA scores range from 0 to 120 points, where higher scores indicate greater LSM. Participants performed the LSA by phone interview.

### 2.4. Accelerometer and GPS Data Collection

Each participant was provided with an accelerometer (Actigraph GT3X+, Actigraph, LLC, Pensacola, FL, USA) and a GPS monitor (QStarz BT-Q1000XT, Qstarz International Co., Ltd., Taipei, Taiwan) to wear for seven consecutive days. The movement sensors were attached around the hip using an elastic belt. The GPS device was set to record at a sample rate of 15 s and the accelerometer at 30 Hz. The accelerometer was initialized in advance using the local computer time. To synchronize the accelerometer and GPS monitor and to minimize potential temporal misalignment, the local computer time was reset to Coordinated Universal Time, using the built-in Windows tool before accelerometer initialization.

Participants were requested to wear the devices during all waking hours for the 7-day period, to document wear time of the devices in an activity diary, to note three frequently visited locations, and to make note of any non-wear periods. The length of this monitoring period differs from the four-week recall period of the LSA. Collection of the LSA, GPS and accelerometer data occurred prior to participants commencing their assigned exercise regimen. Participants were asked to charge the GPS monitor each night. After the measurement period, the participants returned the devices using a prepaid mail service.

### 2.5. Accelerometer and GPS Data Processing

**Figure 1:**
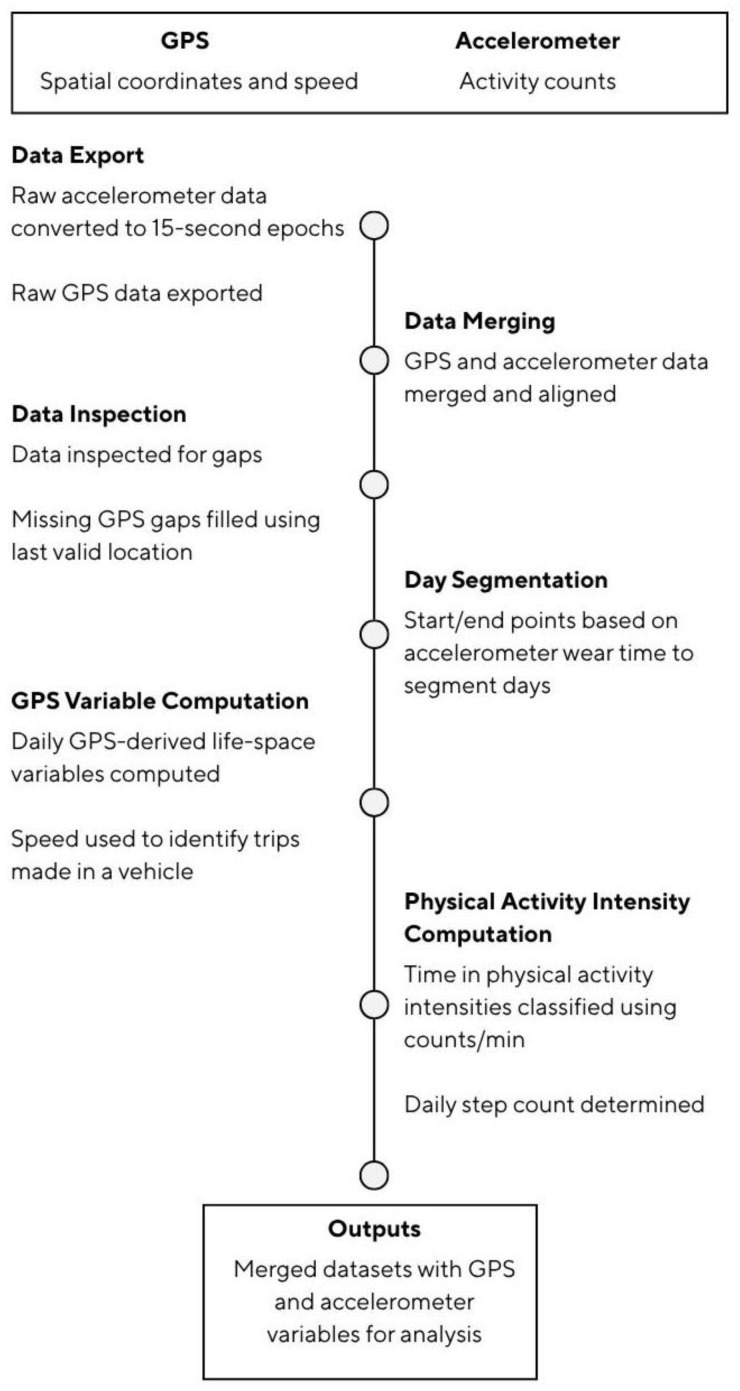
GPS and accelerometer data processing.

The raw accelerometer data were converted to 15-s epochs and exported to excel using the Actilife 6 software version 6.13.4, (Actigraph, LLC, Pensacola, FL, USA). The raw GPS data were exported using the QTravel software version 1.53.000(T), (Qstarz International Co., Ltd., Taipei, Taiwan). GPS and accelerometer data were merged and aligned using Matlab (R2025a, The MathWorks, Natick, MA, USA). GPS measurement often results in large amounts of missing data due factors such as atmospheric conditions, satellite and receiver errors, and multipath errors (i.e., disruptions due to building density) [51], so the data were inspected to identify temporal gaps, and interpolation using the last acceptable GPS point was conducted to produce a complete timeseries of merged daily GPS and accelerometer data [34]. This ensured a continuous record, maximizing the use of synchronized accelerometer data, which without interpolation would have been reduced. Accelerometer wear time was used to determine daily start and end points, allowing the datasets to be separated into individual days. Data processing is outlined in Figure 1.

**Figure 2:**
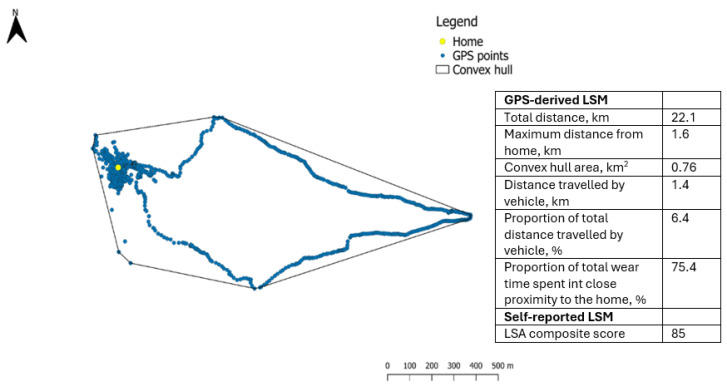
Single-day GPS measurement and derived LSM variables of one participant figure and values reflect a single recording day and are presented for illustrative purposes only.

Daily GPS output variables were computed using QGIS 3.40 (QGIS Development Team), and the GPS-derived life-space variables outlined in Table 1 were exported, as well as figures of the daily continuous GPS trails (Figure 2). Speed was recorded at each GPS time point. Periods with speed ≥10 km/h over at least 90 s were classified as being in a vehicle [34].

Accelerometer raw signals were processed to determine activity levels and step count. Physical activity intensity was classified as follows, sedentary behavior (SB): <100 counts per minute, low-intensity physical activity (LIPA): 100–1040 counts per minute, and moderate-to-vigorous physical activity (MVPA): ≥1041 counts per minute. Non-wear periods were defined as ≥90 consecutive minutes of zero activity counts, allowing for up to 2 min of non-zero count interruption. No minimum weartime threshold was imposed and all days with available wear time were included.

Physical activity performed in close proximity to the home and beyond the home vicinity was investigated by identifying the proportion of time spent in each physical activity intensity level in these two locations. “In close proximity to the home” was defined as any location within a 64-m radius of the home address GPS coordinates. The distance radius for the home environment was determined by examining the distribution of GPS points around the home GPS coordinates on one random day for each participant [52]. The average distribution for GPS points about the home was 64 m, so this buffer size was used to classify the activity occurring in close proximity to the home for all participants. This buffer was intended to encompass all GPS points occurring near to the residence and in the immediate surrounding area.

### 2.6. Statistical Analysis

A pre-analysis plan was published on Open Science Framework (OSF) on 3 November 2025 and updated on 24 November 2025. Data was analyzed using Stata 18 (StataCorp, College Station, TX, USA). Distributions were assessed for normality using the Shapiro–Wilk test, as well as by visually assessing histograms, Q-Q plots, and box and whisker plots. Due to the small sample size, normality testing may not be accurate and so, continuous variables were described using medians and interquartile ranges [53]. Spearman’s correlation coefficients were calculated to examine associations between physical activity outcomes and GPS-derived life-space variables and to examine the association between LSA scores and GPS-derived life-space variables. Wilcoxon signed-rank tests were applied to assess differences in physical activity variables between “in close proximity to the home” and “beyond the home”. The raw *p*-values are reported. Statistical significance was based on false discovery rate (FDR)-adjusted thresholds calculated using the Benjamini–Hochberg procedure.

## 3. Results

### 3.1. Participants

GPS, physical activity, and LSA data were available for 11 participants. Table 2 outlines their characteristics. The participants had a median age of 72 years, and 73% were female. Approximately half of the participants lived in an apartment and half in a house. The median total daily wear time for combined GPS and accelerometer devices was 823 min.

Participants spent approximately two thirds of their daily wear time in close proximity to the home, and they traveled approximately one third of their total distance by vehicle. The self-reported life-space mobility was high, with a median LSA composite score of 82 (Table 3). The individual participant data are provided in Supplementary Table S1, and examples of GPS-derived life-space mobility maps are shown in Supplementary Figure S1.

Spearman’s rank-order correlation was performed to determine the relationship between self-reported life space, as determined by the LSA, and GPS-derived measures of life space (Table 4). Several correlations were observed between the LSA composite score and GPS-derived variables, however none were statistically significant after adjusting for multiple comparisons.

### 3.2. Location-Specific Physical Activity and Life-Space Measurements

**Figure 3:**
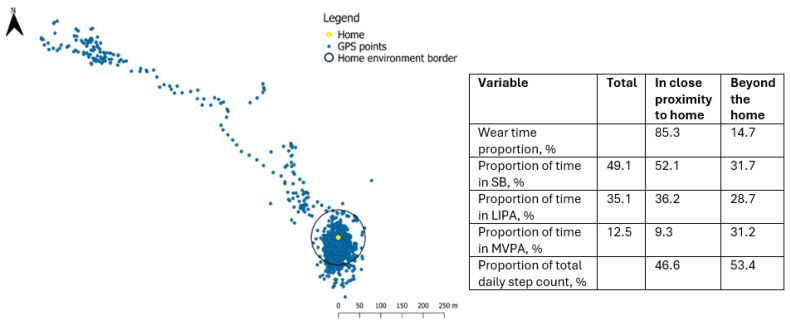
Single-day GPS points and physical activity intensities in close proximity to the home and beyond the home environment of one individual figure and values reflect a single recording day and are presented for illustrative purposes only. Proportions of physical activity intensities in close proximity to the home and beyond the home are expressed relative to wear time in each location.

The majority of the accelerometer/GPS wear time was spent sedentary (median = 532 min/day). This was followed by LIPA (median = 146 min/day), and MVPA (median = 68 min/day) (Table 1). The distribution of GPS points in close proximity to the home and beyond the home is presented in one participant in Figure 3. The GPS points in close proximity to the home are shown within the circle.

The proportion of time spent in each physical activity intensity, by location, is shown in Table 5. Participants spent a higher proportion of time sedentary in close proximity to the home (median 77.8%) compared to beyond the home (median 36.6%), whereas the proportion of time spent performing moderate-to-vigorous physical activity and the proportion of total daily step count were higher beyond the home (medians 12.9% and 67.8%, respectively) compared to in close proximity to the home (medians 5.2% and 30.3%, respectively).

Spearman’s rank-order correlations were conducted to investigate associations between physical activity variables and GPS-derived life-space outcomes. Associations were weak, and no significant correlations were identified (ρ range: −0.26 to 0.45, all *p* > 0.05).

### 3.3. Health-Related Quality of Life and Life-Space Measurements

Health-related quality of life demonstrated weak associations with the LSA. Correlations were observed between some GPS-derived life-space measures and measures of health-related quality of life (Table 6). No associations were noted between PDQ-39 scores and any life-space measurements. Better EQ-5D scores were associated with greater LSM. However, neither of these associations remained statistically significant after adjusting for multiple comparisons.

## 4. Discussion

The purpose of this study was to examine the LSM of people with mild-to-moderate PD, to explore the agreement between self-reported and objective measures of LSM, and to assess physical activity and health-related quality-of-life outcomes in relation to LSM in this cohort. A combination of GPS and accelerometer data as well as self-reported LSA data was collected at baseline measurement in a sub-cohort of participants enrolled in a larger RCT to fulfill these aims.

Participants in this study demonstrated high levels of inter-individual variability in GPS-derived LSM. Mobility and life space are determined by a combination of several factors [54], so this variability may reflect factors such as environmental differences, personal capacity or preference, and accessibility to transport methods. The extent of GPS-derived life space in this cohort is broad and dispersed, with high total distances traveled, and large areas covered daily. While there is limited literature regarding GPS-measured life space in people with PD, the results from this study mirror, or exceed, values identified in community-dwelling older adults, suggesting that LSM in this cohort may be comparable to values reported in non-PD populations [38,55,56].

High levels of self-reported LSM (LSA scores) were documented in this study, suggesting that participants engaged in their wider community and environment. Previous investigations using the LSA in people with PD have identified lower life-space mobility, indicating perhaps that the cohort in this study is a highly motivated and mobile group [29,32,33]. Additionally, high LSA scores have been captured in studies including high-functioning community-dwelling older adults with PD [30], and participants enrolled in an RCT aimed at improving walking ability [31], supporting the effect that individual motivation has on self-reported LSM. Although ceiling effects of the LSA have previously been noted [30], these findings may suggest that the instrument is capable of capturing the large extent of LSM in people with PD with a high level of functioning, without reaching a ceiling effect, although further investigation is warranted.

This cohort demonstrated high levels of self-reported LSM despite considerable variability in GPS-derived measures, suggesting that individuals may maintain interaction with the extended environment and community, but do so through distinctly different mobility patterns. Previous research similarly identified wide variation in GPS-derived measures of life space among individuals with the same LSA score [30], indicating that the LSA outlines perceived life space while GPS measures outline the specific patterns of movement. Echoing prior research [36], positive correlations between self-reported and objective measures were observed, however these associations were not statistically significant after correction for multiple comparisons. This highlights potential conceptual overlap between the measures, while also suggesting that these approaches may capture different aspects of mobility. However, given the small sample size and non-significant results, these observations remain exploratory.

Physical activity has beneficial effects on motor signs and quality of life in people with mild-to-moderate PD [57,58]. Capturing the extent and location of where physical activity occurs could allow for more targeted strategies for implementing beneficial physical activity interventions in this population. In keeping with previous literature, participants in this study spent a high proportion of their time in close proximity to the home being sedentary, whereas the median proportion of time spent in MVPA (12.9% vs. 5.2%) and proportion of total daily step count (67.8% vs. 30.3%) were higher beyond the home environment than in close proximity to the home [39,42]. Awareness of the influence of location on physical activity levels can allow practitioners to support the reduction of sedentary behavior in close proximity to the home, while also promoting moderate-intensity physical activity by encouraging movement outside the home environment. This knowledge may assist in goal setting and support a more person-centered approach to physical activity interventions.

The use of digital mobility outcomes in various cohorts of older adults is continuously expanding, and with that comes progression of their implementation, analysis, and reporting. Although GPS and accelerometry have been used in combination previously [34,39,42,59], there is a lack of consistency and guidance in the analysis and reporting of these methods. There is a need for clearly outlined methodology and so, this research aimed to systematically manage and describe the processes undertaken in collecting, processing, and analyzing the GPS and accelerometer data. While there is always potential for development, this study outlines one possible approach in the use of these specific digital mobility outcomes. It must be acknowledged that combined GPS and accelerometer data will contain inconsistencies, however in the opinion of the authors, these inconsistencies were addressed in the most appropriate manner for this dataset.

Several clinical implications have emerged from this research. Although GPS monitors can provide more detailed information regarding an individual’s LSM, they are currently best suited as a complement to self-reported measures. The LSA may capture the overall perceived extent of an individual’s movement in this cohort, with GPS providing supplemental spatial and contextual detail. Where GPS measurements may be of more benefit is in terms of location-based physical activity. These findings are exploratory, but understanding where an individual is going and what activity they are doing may help practitioners to better target their strategies, potentially resulting in more specific, and potentially beneficial outcomes in physical activity interventions. Tools such as the Participation in ACTivities and Places OUTside Home (ACT-OUT) questionnaire [60] may provide additional information regarding locations visited or abandoned, complementing objective mobility data. These findings may also suggest that moving beyond the home vicinity shows higher levels of MVPA and step count, which could inform strategies aimed at encouraging improvements in these physical activity behaviors. However, mobility behavior is multifactorial [54], comprising both motor and cognitive function, and while identifying where individuals are most active is informative, consideration should be given to other contextual factors to generate a holistic approach to LSM and physical activity.

This study has several strengths and limitations. The combined use of self-reported and objective measures of LSM presents an in-depth understanding of participants’ mobility patterns. Additionally, the systematic collection, processing, and analysis of GPS and accelerometer data provides a transparent methodological approach that can guide future research. However, GPS and accelerometer data are subject to inconsistencies, although these were addressed as appropriately as possible in the current analysis, processing methods may have prolonged stationary periods and influenced mobility outcomes. The proportion and duration of signal gaps were not formally quantified, and sensitivity analyses without interpolation were not conducted. Another limitation is that the study involved a relatively small sub-cohort of participants enrolled in a physical activity intervention study, which may limit generalizability to the broader PD population. It is important to consider that this is a group who actively engaged in a physical activity intervention and so it can be speculated that a lower LSM may be demonstrated in other cohorts of the population. Given the small sample size, the findings of this work should be interpreted as preliminary and exploratory. A further consideration is the difference in assessment periods between measures. The LSA reflects mobility over the previous four weeks, whereas accelerometer and GPS data were collected over a single week. The accelerometer and GPS monitoring period is intended to reflect habitual activity, however week-to-week variability may influence agreement between subjective and objective measures. Activity monitoring was performed without detailed consideration of medication timing, motor fluctuations, or walking aid use. The 64-m radius used to define activity in close proximity in the home was derived from GPS distributions on a single day for each participant from a relatively small sample. As such, the radius is somewhat larger than some previously reported values [43], which may influence the classification of activities occurring near the home. This radius was applied uniformly to all participants, however given the variability in housing sizes it is possible that this approach may have produced minor misclassification of indoor versus outdoor activity. The use of standard adult accelerometry thresholds along with a lack of minimum weartime requirement due to our small sample size may influence the precision of physical activity outcomes. These factors may influence daily activity patterns so findings should be interpreted with caution.

Maintaining a broad LSM may support beneficial physical activity behaviors, perhaps contributing to sustained or improved quality of life. Future research should consider the longitudinal effects on life space and physical activity in this population in order to determine the benefits and necessity of sustaining LSM, and further large-scale investigations would help to strengthen understanding of the findings and the implications of this research. Additionally, extending spatial categorizations past “in close proximity to the home” and “beyond the home”, for example “within the neighborhood”, may be of benefit in providing further contextual understanding of mobility and activity levels in this population.

## 5. Conclusions

The combination of digital and self-reported measures of life space utilized in this study provides preliminary insight into the extent of life-space mobility among people with mild–moderate PD. Participants demonstrated a diverse spread of life space with high levels of variability through both the self-reported LSA and GPS-derived measures of life space. Physical activity levels differed between locations, with sedentary behavior higher in close proximity to the home, and MVPA and step count higher beyond the home. Given the small sample size, these findings should be interpreted as preliminary and exploratory. Knowing patterns and locations of physical activity behaviors may inform strategies for reducing SB and improving physical activity levels in this group. In this context, GPS-derived measures of life space may provide a useful complement to self-reported life-space measures.

## Figures and Tables

**Table 1:** GPS-derived life-space variables.

Variable	Description
Total distance, km	Cumulative distance traveled, calculated as the sum of the distances between consecutive GPS points
Maximum distance from home, km	Straight-line distance from the home to the furthest GPS point recorded
Convex hull area, km^2^	Area of the smallest polygon that encompasses all recorded GPS points
Distance traveled by vehicle, km	Sum of distances between GPS points where speed was ≥10 km/h for at least 90 s

**Table 2:** Participant characteristics.

Characteristic	n = 11
Age, y	72 (66, 77)
Gender, n (%)	
Female	8 (72.7)
Male	3 (27.3)
Height, cm	167 (160, 172)
Weight, kg	65 (55, 85)
Years since PD diagnosis	6 (3, 9)
Hoehn and Yahr stage, n (%)	
2	9 (81.8)
3	2 (18.2)
Have fallen in previous 6 months, n (%)	3 (27.3)
Use a walking aid outdoors, n (%)	3 (27.3)
Housing type, n (%)	
Apartment	6 (54.5)
House/villa	5 (45.5)
Marital status, n (%)	
Single	2 (18.2)
Cohabiting	9 (81.8)
Education, y	15 (13, 16)
5xSTS, s	10.9 (10.1, 12.6)
Mini-BESTest	22 (20, 23)
Walk-12	11 (3, 17)
PDQ-39 summary index	13.2 (5.7, 22.9)
EQ-5D	
Index	0.75 (0.59, 0.8)
VAS, %	70 (60, 80)
ABC scale, %	90 (66.3, 94.2)
Total daily accelerometer/GPS wear time, min	823.3 (734.1, 926.7)
SB, min/day	532.0 (417.0, 673.0)
LIPA, min/day	145.8 (120.2, 271.5)
MVPA, min/day	67.9 (32.2, 97.0)
Step count, steps/day	5400 (3010, 7273)

Variables are presented as median (Q1, Q3) unless otherwise stated. The Five Times Sit-to-Stand (5xSTS) test: lower scores indicate better lower-limb functional performance. The Mini Balance Evaluation Systems Test (Mini-BESTest): 0–28; higher scores indicate better dynamic balance performance. Walk-12: 0–42; higher scores indicate greater perceived walking difficulty. The Parkinson’s Disease Questionnaire-39 (PDQ-39) summary index: 0–100; higher scores indicate worse quality of life. The Euroqol 5-Dimensions scale (EQ-5D) Index: 0–1; higher scores indicate better health. EQ-5D Visual Analog Scale (VAS): 0–100; higher scores indicate better self-rated health. Activity-specific Balance Confidence scale (ABC): 0–100%; higher scores indicate greater balance confidence.

**Table 3:** GPS-derived and self-reported life space.

Variable	n = 11
Total distance, km	26.3 (19.3, 42.6)
Maximum distance from home, km	5.3 (1.3, 18.1)
Convex hull area, km^2^	8.7 (0.6, 89.1)
Distance traveled by vehicle, km	10.6 (3.5, 27.6)
Proportion of total distance traveled by vehicle, %	30.9 (20.9, 53.5)
Proportion of total wear time spent in close proximity to the home, %	62.3 (40.6, 76.0)
LSA composite score	82 (54, 94)
LS-M	5 (4, 5)
LS-E	5 (4, 5)
LS-I	5 (2, 5)

Continuous variables are presented as median (Q1, Q3). LSA composite score: 0–120; higher scores indicate greater LSM. LS-M: the highest life-space level attained even if equipment or help from a person was used: 0–5. LS-E: the highest life-space level attained without help from a person: 0-5-LS-I: the highest life-space level attained without help from a person and without using any equipment: 0–5.

**Table 4:** Spearman correlations between self-reported and GPS-derived life space.

Self-Reported Variable	GPS-Derived Variable	ρ	95% CI	*p*-Value	Adjusted *p*-Value
LSA composite score	Total distance, km	0.67	0.21, 1.13	0.03	0.12
	Maximum distance, km	0.46	−0.19, 1.10	0.16	0.21
	Convex hull, km^2^	0.41	−0.19, 1.01	0.21	0.21
	Distance in vehicle, km	0.47	−0.08, 1.03	0.14	0.28

Spearman correlation coefficients are presented with 95% confidence intervals. FDR-adjusted *p*-values shown.

**Table 5:** Comparisons of physical activity variables in close proximity to and beyond the home environment.

Variable	Total	In Close Proximity to the Home	Beyond the Home	95% CI	Analysis	Adjusted *p*-Value
Location-based weartime proportion, %		62.4 (47.1, 79.5)	37.6 (20.5, 52.9)	2.0, 49.0	z = 1.87, *p* = 0.06	0.08
Proportion of time in SB, %	72.5 (51.9, 78.0)	77.8 (55.3, 86.2)	54.7 (28.4, 70.1)	7.9–39.8	z = 2.93, *p* < 0.01	0.02 *
Proportion of time in LIPA, %	18.5 (13.5, 33.8)	22.7 (12.5, 34.2)	19.8 (17.9, 33.3)	−4.8, 4.0	z = −0.36, *p* = 0.72	0.72
Proportion of time in MVPA, %	7.5 (3.9, 13.2)	5.2 (1.9, 8.7)	12.9 (8.3, 32.1)	−24.9, −4.6	z = −2.85, *p* < 0.01	0.04 *
Proportion of total daily step count, %		30.3 (24.3, 41.5)	67.8 (58.5, 73.8)	−46.5, −19.9	z = −2.93, *p* < 0.01	0.02 *

Proportions of physical activity intensities in close proximity to the home and beyond the home are expressed relative to wear time in each location. Continuous variables are presented as median (Q1, Q3). Differences between within- and beyond-home values were calculated and are reported with 95% confidence intervals (CI). FDR-adjusted *p*-values are shown. * indicates statistical significance after FDR correction. SB: Sedentary behavior. LIPA: Low-intensity physical activity. MVPA: Moderate-to-vigorous physical activity.

**Table 6:** Spearman correlations between self-reported measures of health-related quality of life and life-space measures.

Health-Related Quality of Life	Life-Space Measurement	ρ	95% CI	*p*-Value	Adjusted *p*-Value
PDQ-39	Total distance, km	−0.12	−0.90, 0.67	0.75	1
	Maximum distance, km	−0.14	−0.93, 0.65	0.70	1
	Convex hull, km^2^	−0.22	−1.04, 0.59	0.53	1
	Distance in vehicle, km	0.04	−0.75, 0.84	0.91	0.91
	LSA	−0.06	−0.84, 0.72	0.87	1
EQ-5D Index	Total distance, km	0.41	−0.24, 1.07	0.20	0.33
	Maximum distance, km	0.46	−0.22, 1.14	0.15	0.38
	Convex hull, km^2^	0.67	0.16, 1.18	0.03	0.15
	Distance in vehicle, km	0.40	−0.22, 1.03	0.22	0.28
	LSA	0.23	−0.54, 0.99	0.50	0.50
EQ-VAS	Total distance, km	0.71	0.19, 1.22	0.02	0.1
	Maximum distance, km	0.50	−0.08, 1.09	0.11	0.14
	Convex hull, km^2^	0.56	0.03, 1.09	0.07	0.18
	Distance in vehicle, km	0.57	0.03, 1.11	0.07	0.12
	LSA	0.34	−0.37, 1.04	0.31	0.31

Spearman correlation coefficients are presented with 95% confidence intervals. FDR-adjusted *p*-values are shown. PDQ-39: The Parkinson’s Disease Questionnaire-39, summary index. EQ-5D Index: The Euroqol 5-Dimensions scale Index. EQ-VAS: The Euroqol 5-Dimensions Visual Analog Scale.

## Data Availability

The datasets presented in this article are not readily available because the dataset contains personally identifiable information and is therefore subject to ethical and legal restrictions on public sharing, according to Swedish laws. Requests to access the datasets should be directed to the corresponding author.

## Supplementary Materials

The following supporting information can be downloaded at: https://www.mdpi.com/article/10.3390/s26082480/s1, Table S1. Participant 3 GPS-derived and self-reported life-space. Figure S1. Examples of individual participant life-space mobility maps.

## Abbreviations

The following abbreviations are used in this manuscript:

ABC	Activity-Specific Balance Confidence Scale
ACT-OUT	Participation In Activities And Places Outside Home
EQ-5D	Euroqual 5-Dimension Scale
GPS	Global Positioning System
ICF	International Classification of Functioning, Disability and Health
LIPA	Low-Intensity Physical Activity
LSA	Life-Space Assessment
LSM	Life-Space Mobility
LS-E	Highest LSA Life-Space Level Attained Without Help From a Person
LS-I	Highest LSA Life-Space Level Attained Without Help From a Person and Without Using Any Equipment
LS-M	Highest LSA Life-Space Level Attained Even if Equipment or Help From a Person Was Used
Mini-BESTest	Mini Balance Evaluation Systems Test
MoCA	Montreal Cognitive Assessment
MVPA	Moderate-To-Vigorous Physical Activity
OSF	Open Science Framework
PD	Parkinson’s Disease
PDQ-39	Parkinson’s Disease Questionnaire-39
RCT	Randomized Controlled Trial
SB	Sedentary Behavior
STEPS	Support for Home Training using Ehealth in Parkinsons Disease
STS	Sit-To-Stand
VAS	Visual Analog Scale
