# Supplementary material for: Integrating GPS and Accelerometry to Capture Life-Space Mobility in Parkinson’s Disease
Source: Sensors (Basel). 2026 Apr 17;26(8):2480. doi: 10.3390/s26082480 (PMC13119923; doi:10.3390/s26082480)
Supplement: Supplementary file 1 [file sensors-26-02480-s001.zip › supplementary maps.pdf]

Figure S1. Examples of individual participant life-space mobility maps.

Participant 1

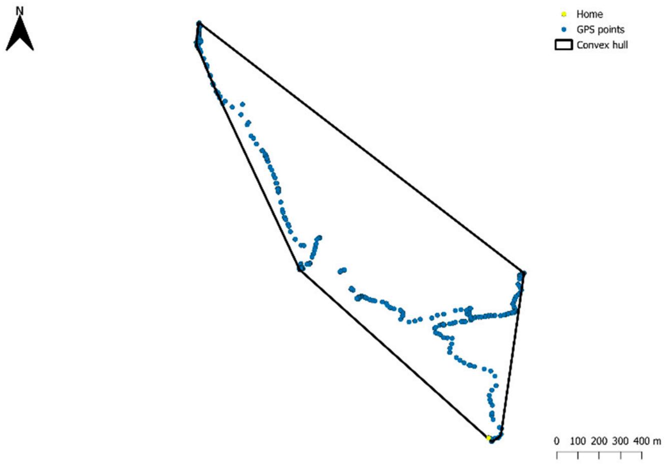

Participant 3

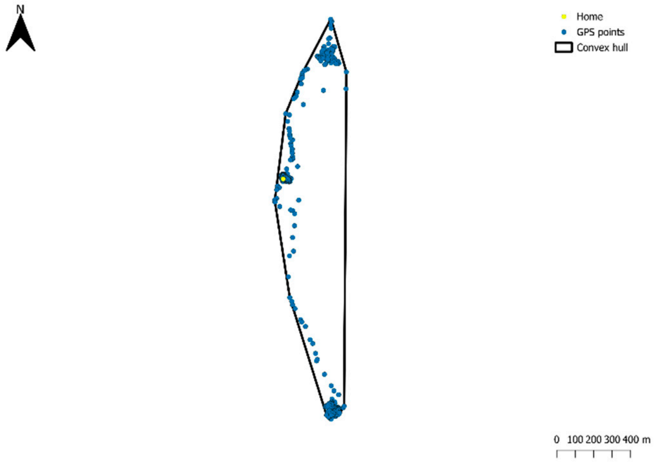

Participant 8

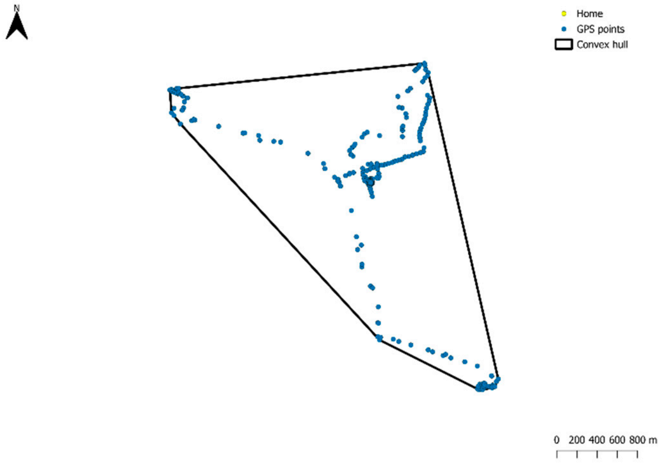

Examples of GPS-derived life-space mobility maps from three participants illustrating variation in spatial mobility patterns. Maps are presented for illustrative purposes. Maps for all participants are not shown.
