# Supplementary material for: Integrating GPS and Accelerometry to Capture Life-Space Mobility in Parkinson’s Disease
Source: Sensors (Basel). 2026 Apr 17;26(8):2480. doi: 10.3390/s26082480 (PMC13119923; doi:10.3390/s26082480)
Supplement: Supplementary file 1 [file sensors-26-02480-s001.zip › supplementary table.pdf]

Table S1. Participant 3 GPS-derived and self-reported life-space

**Participant ID**	**Total distance, km**	Maximum distance from home, km	Convex hull area, km^2	Distance travelled by vehicle, km	Proportion of total distance travelled by vehicle, %	Proportion of total wear time spent within the home, %	LSA composite score	LS-M	LS-E	LS-I
1	74.61	20.75	140.27	54.78	74.03	32.45	74	5	5	5
2	32.90	5.51	8.14	18.37	53.51	62.33	54	4	4	2
3	13.55	1.32	0.57	3.21	23.67	81.66	48.5	5	5	2
4	20.63	3.41	8.73	10.63	38.86	75.97	82	5	5	5
5	18.92	1.00	0.52	3.54	20.13	70.72	35	4	4	1
6	19.52	1.11	0.55	1.80	9.33	69.91	85	5	4	4
7	41.91	4.73	6.27	9.55	20.94	40.23	96	5	5	5
8	26.28	5.26	21.26	20.32	57.14	87.37	94	5	5	5
9	57.21	21.34	89.14	35.93	44.73	53.87	100	5	5	5
10	42.59	18.05	128.01	27.61	48.22	46.38	94	5	5	5
11	19.33	6.64	23.34	9.34	30.87	40.62	54	4	4	4

Life-Space Assessment (LSA) composite score: 0-120, higher scores indicate greater life-space mobility. LS-M: the highest life-space level attained even if equipment or help from a person was used: 0-5. LS-E: the highest life-space level attained without help from a person: 0-5- LS-I: the highest life-space level attained without help from a person and without using any equipment: 0-5.
